# Efficient Absorbance-Based Assay for Rapid Antibiotic Susceptibility Testing of Enterobacterales

**DOI:** 10.3390/antibiotics13090852

**Published:** 2024-09-06

**Authors:** Carolina Axelsson, Bo Nilson, Ann-Sofi Rehnstam-Holm

**Affiliations:** 1 Department of Environmental Science and Biomedical Science, Faculty of Natural Science, Kristianstad University, 288 91 Kristianstad, Sweden; ann-sofi.rehnstam-holm@hkr.se; 2 Department of Clinical Microbiology, Office for Medical Services, Region Skåne, 223 62 Lund, Sweden; bo.nilson@med.lu.se; 3 Division of Medical Microbiology, Department of Experimental Medicine, Faculty of Medicine, Lund University, 221 84 Lund, Sweden

**Keywords:** antibiotic, resistance, susceptibility, Enterobacterales, absorbance, spectrophotometry

## Abstract

It is increasingly important to rapidly receive information on the antimicrobial susceptibility of bacteria due to the rise in antimicrobial resistance worldwide. However, traditional phenotypic methods are time-consuming. Thus, the objective of this study was to develop a rapid assay that can detect antibiotic-resistant bacterial isolates phenotypically in less than 2 h. The microplate assay used in this study is based on absorbance measurements of pure bacterial isolates grown in liquid media with and without antibiotics. Absorbance was measured at the beginning of the assay and after 90 min of incubation at 37 °C. Susceptibility was calculated for bacterial isolates that, in the absence of antibiotics, exhibited more than a 50% growth increase by comparing the absorbance value of the culture in the presence of an antibiotic at 90 min with its initial value. Here, we show that it is possible to phenotypically screen the antibiotic susceptibility of Enterobacterales and *Acinetobacter* spp. isolates to three different antibiotics in 90 min. The test demonstrated an accuracy of 98.8%, sensitivity of 97.6%, and specificity of 99.6%. The false susceptibility rates were 0.2% and false resistance rates were 1.0%. This rapid and simple absorbance test has proven suitable for the screening of antibiotic susceptibility for a large number of strains with high accuracy and sensitivity. This method can be performed without specialized and costly materials and/or equipment, which makes it highly suitable for laboratories with limited resources.

## 1. Introduction

Antimicrobial resistance is a significant global concern and infections caused by antimicrobial-resistant pathogens pose serious threats to both humans and the biosphere. The rapid, simple, cost-efficient, and reliable screening of antibiotic susceptibility is crucial in order to reduce the emergence and spread of resistant pathogens and to use existing antimicrobial therapies more effectively [1]. Indeed, if not controlled, infections caused by antibiotic-resistant bacteria are estimated to result in 10 million deaths annually and the cost of this might reach USD 100 trillion by 2050 [2]. The global pandemic of antibiotic resistance demands new solutions for the faster and more accurate detection of antimicrobial susceptibility and also highlights the importance of reducing antibiotic usage. Therefore, it is crucial to have antibiotic susceptibility assays that are easy to perform and cost-effective, especially in resource-limited settings. Standard clinical laboratory detection methods like disc diffusion, E-test gradient diffusion, and broth dilution susceptibility tests rely on culturing techniques conducted using pure isolates [3]. Despite being time-consuming, these methods are widely used due to their low cost and ease of use. Since these methods are slow, with answers normally generated after 18–36 h, they are unsuitable for the screening of large numbers of isolates and not fast enough to generate life-saving information in severe cases of infections. The analytical timeframe can be shortened using commercial phenotypically based micro-plate systems such as MicroScan (Siemens Healthcare Diagnostics GmbH, USA), Phoenix (BD Diagnostic Systems, USA), or Vitek 2 (bioMérieux, France), which can provide susceptibility results for pure cultures of fast-growing bacteria in approximately five to eight hours [4]. Other kinds of rapid phenotypically based methods are different agglutination tests for specific resistance which can be performed within 30 min, or tests based on enzymatic activity, which can generate results in 15 min [5]. In addition, several other rapid tests, like Raman spectroscopy [6], mother machine-based analyses [7,8], single growth analyses [9], and impedance-based electro-microfluidic devices [10], have been established. However, most of these tests require specific kits and/or special equipment that are often restricted to their specific use and are usually expensive. 

Antibiotic susceptibility tests based on molecular diagnostic techniques deliver results faster than traditional phenotypically based methods. DNA-based methods, such as polymerase chain reaction (PCR) or other nucleic acid-detection-based methods are useful and fast, with results generated within 1/2–2 h. In addition, pure cultures or even culturing is not always required, in contrast to the commonly used phenotypical tests. However, molecular methods are restricted to the detection of currently known resistance-associated genetic loci. This is especially problematic in extended-spectrum β-lactamase (ESBL)- and carbapenem-resistant strains, since several hundred genes can cause these phenotypes in Enterobacterales [11,12], a fact that has restricted the general use of these methods when a large number of bacterial isolates have to be screened at low costs. Antibiotic resistance genes are also under constant evolution, which means that information regarding the bacterial phenotype of antibiotic resistance is far more important than the genotype [13]. The detected gene fragments in PCR systems might also originate from pathogens already suppressed or killed by antibiotics, or fragments whose origin is unknown [14,15]. Other susceptibility tests that are under development include mass spectrometry, next-generation-sequencing-based methods, and several other alternative technologies like electronic nose devices and vibrational- and absorption-based spectroscopy methods [16]. All these methods have, however, not been implemented in clinical laboratories and will probably never be applicable to small laboratories or in laboratories with limited funding. 

In this study, we evaluated the antibiotic susceptibility of >300 isolated environmental and clinical strains of Enterobacterales and *Acinetobacter* spp. by following the absorbance differences in strains grown in liquid medium with or without the antibiotics cefotaxime (CTX); meropenem (MER); and ciprofloxacin (CIP). We used a common microplate reader, available in most laboratories, which means that the method can easily be used at a low cost. 

## 2. Results

**Figure 1:**
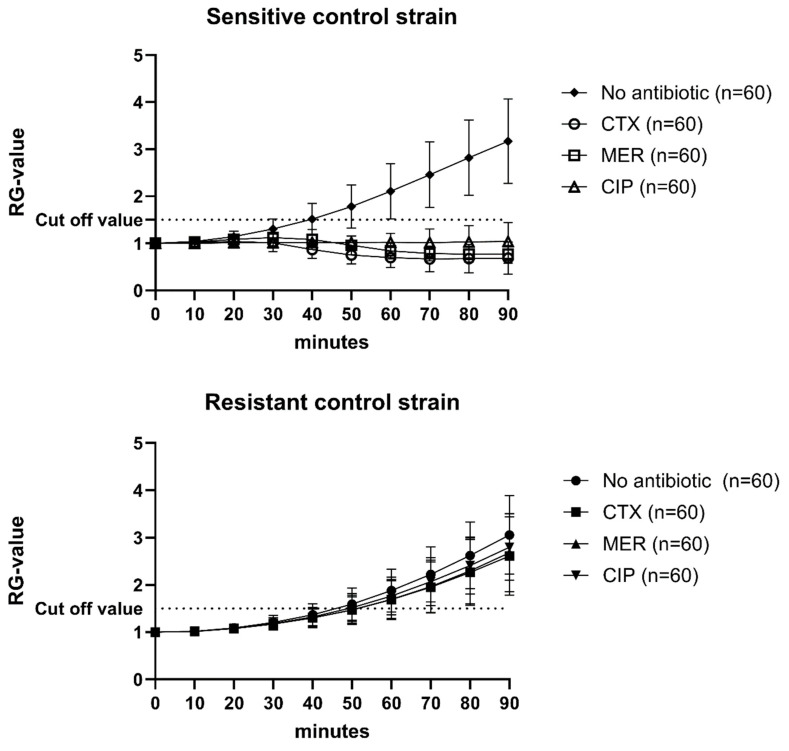
Mean RG values and standard deviations of 60 individual analyses of sensitive and resistant control strains. CTX = cefotaxime; MER = meropenem; CIP = Ciprofloxacin. Cut-off value was set to 1.5.

Every single assay included a positive and a negative control for the three antibiotics, as well as an antibiotic-free growth control (*n* = 60; Figure 1). The Relative Growth value (RG value) was calculated by normalizing the absorbance values for every time point using the absorbance value at 0 min. The cut-off value was set to 1.5, i.e., 50% growth (Figure 1). If the controls, as well as the isolates, did not grow by at least 50% in wells without antibiotics, the assays were re-analyzed.

Overall, all bacterial isolates (*n* = 366) analyzed, irrespective of origin and cultivation method, showed an accuracy of 98.8% with a sensitivity of 97.6% and a specificity of 99.6 (number of individual assays = 2996; Table 1). The Very major error (false susceptibility) rate was 0.2% and the Major error rate (false resistance) was 1.0%. The highest error rates were recorded for ciprofloxacin (1.6%).

The analysis that only included the control strains gave an overall accuracy of 99.2%, with the highest Major error values for cefotaxime and ciprofloxacin (Table 2). The analysis that only included the environmental and clinical strains (Table 2) gave an overall accuracy of 96.9%, with the highest error rate for ciprofloxacin (2.2%).

## 3. Discussion

We here demonstrated a method that had an overall sensitivity of 97.6%, specificity of 99.6%, and accuracy of 98.8% against three tested antibiotics. The Very major error and Major error rates of the control strains were 0% and 1.0%, respectively. The corresponding results were 0.3% and 1.0% for the clinical and environmental isolates. This is well within the same range as, or even better than, that of many previous studies performed using automated phenotypical systems on Enterobacterales [3,11,17,18,19,20]. The recorded error rate for cefotaxime was 1.3% (0.4% Very major errors and 0.9% Major errors). A large majority of the environmental isolates were previously verified as ESBL strains, both genetically and phenotypically. Therefore, these Major errors cannot be explained by phenotypical or genotypical differences in the isolates. The highest error rates were recorded in the clinical isolates for ciprofloxacin (1.9%). This was mainly due to problems related to the analyses of strains directly collected from blood cultures. This phenomenon has previously been confirmed when using the ASTRA method [21,22]. 

We conducted the assay in duplicate to ensure reliable and reproducible results. In cases where discrepancies were observed between duplicates, the assay was repeated to verify the findings. This approach allowed us to maintain accuracy while balancing the practical constraints of the assay. Rigorous controls and validation steps were also incorporated into every assay to ensure the robustness of the data. 

The method proposed in this study differs from the conventional microdilution broth method in several key aspects, offering distinct advantages, particularly in terms of speed and efficiency. First, while the conventional microdilution method typically requires multiple concentrations of antibiotics to determine the minimum inhibitory concentration (MIC) [23], our method uses fixed concentrations of antibiotics. This allows for the rapid screening of resistance across multiple strains on the same assay plate, significantly reducing the time required for analysis. Second, the conventional microdilution method generally requires 16 to 24 h of incubation before results can be read, as it relies on visible growth inhibition. In contrast, our method is designed to yield results within just 90 min, providing a much faster assessment of antibiotic susceptibility. This rapid turnaround time is particularly advantageous where timely decisions are crucial or when large numbers of isolates must be screened during a limited timeframe. Thus, by focusing on a quicker, more streamlined approach, our method enables the analysis of multiple bacterial strains simultaneously, making it a practical and efficient tool for high-throughput screening. The limitation of this method is the need for pure isolates and species that have a ≤40-min generation time. However, this is a very common feature of most Gram-negative pathogens belonging to Enterobacterales or *Acinetobacter* spp.

When applying this method to samples, especially from clinical or environmental sources containing potentially multiple strains, it is crucial to first culture and isolate individual colonies for accurate testing. Although the assay can detect the dominant strain in samples like blood cultures, which is always the primary cause of infection, further steps, such as sub-culturing, are necessary when multiple strains are present to ensure precise antimicrobial susceptibility testing.

If not controlled, infections caused by antibiotic-resistant bacteria will threaten the achievements of modern medicine and result in a post-antibiotic era in which common infections can lead to life-threatening conditions and death [24,25]. This global antibiotic resistance pandemic not only demands new solutions to generate better and faster methods to detect antimicrobial susceptibility, but it also points to the importance of decreasing antibiotic usage and misusage. The lack of regulations is especially severe in developing countries, where most antimicrobials can usually be purchased without medical prescription [26]. The shortage of effective and reliable surveillance and updated information regarding the antibiotic-resistant bacteria within a population increases the problem. Small laboratories are in need of low-cost, easy to perform, and time-saving methods [27]. The assay described here, which can verify the resistance to three antibiotics of up to 100 isolates a day, can easily be adapted to laboratories with limited resources and might be especially important for screening the antibiotic susceptibility of bacteria in laboratories situated in low-income countries.

## 4. Materials and Methods 

### 4.1. Bacterial Strains

Reference strains (*n* = 15) with resistance or susceptibility to the analyzed antibiotics, cefotaxime, meropenem, and ciprofloxacin, were obtained from the Culture Collection University of Gothenburg (CCUG), Sweden; American Type Culture Collection (ATCC); and Culture Collection Department of Clinical Microbiology, Region Skåne (CMRS), Lund, Sweden (Table 3). Clinical isolates (*n* = 45) were also obtained from CMRS. All patient isolates were either *Escherichia coli* (*n* = 38), *Klebsiella. pneumoniae* (*n* = 6), *Enterobacter cloacae* (*n* = 1), or *Citrobacter freundii* (*n* = 1).

Environmental bacterial strains (*n* = 320) were isolated from water samples from Helge Å River, Kristianstad, Sweden, during 2014–2023. All environmental isolates were originally isolated on ESBL agar plates (ESBL ChromAgar, Biomérieux, Lyon, France, containing cefotaxime) and re-cultured on Brain Heart Infusion agar plates (Oxoid Ltd., Basingstoke Hampshire, UK) to obtain pure cultures. The isolates were identified to their species level by MALDI-TOF MS (Bruker, Billerica, MA, USA) and stored in Brain Heart Infusion Broth (Sigma Aldrich, Saint Louis, MO, USA) with 15% glycerol at −25 °C before use in the assay. The strains were identified as belonging to *E. coli* (*n* = 179), *Klebsiella* spp. (*n* = 57), other Enterobacterales (*n* = 75), and *Acinetobacter* spp. (*n* = 9).

### 4.2. Antibiotic Susceptibility Analyses Using Disc Diffusion or E-Strip Tests

The antibiotic resistance of the isolates was identified according to the European standard (EUCAST) routine diagnostic protocols, using disc diffusion or E-strip tests [23]. This was performed in order to determine and demonstrate the specific susceptibility of the isolates or the MIC value of the given antibiotics CTX, MER, and CIP for each bacterial isolate. In brief, a few bacterial colonies were suspended in 0.85% sterile NaCl to a turbidity of 0.5 McFarland standards. This bacterial suspension was spread evenly on to Müller Hinton Agar plates (Sigma Aldrich, Saint Louis, MO, USA) using sterile cotton swabs. E-test strips (Oxoid Ltd., Basingstoke Hampshire, UK) or antibiotic filter discs (Oxoid Ltd., Basingstoke Hampshire, UK) were placed on to the inoculated agar plates, which thereafter were incubated at 37 °C for 18–22 h. Inhibition zones in the disc diffusion test were measured and susceptibility identified according to the EUCAST tables for the given species. In the E-strip test, minimal inhibition concentration (MIC) values were read from the scale on each individual strip in terms of µg/mL.

### 4.3. Bacterial Preparation 

The environmental isolates and the control strains where precultured on Müller Hinton agar before the analysis and were suspended in pre-warmed (37 °C) Müller Hinton Cation Adjusted Broth (MHCA, Sigma-Aldrich, Saint Louis, MO, USA) to McFarland 1.0 before the analysis. 

Clinical strains were obtained from positive blood cultures. Reference strains (Table 3) were inoculated into blood culture bottles (BD Bactec Plus Aerobic/F Culture and BD Bactec Plus Anaerobic Lytic/F) and incubated in an automated BACTEC FXTM blood culture system (Becton Dickinson, Franklin Lakes, NJ, USA) until flagged as positive when the clinical samples were analyzed. The preparation of the bacteria was as described in Axelsson et al. (2019) [21]. Briefly, 1 mL of blood from the aerobic bottles was added to 200 µL of Saponin (5%) and 1 mL of the mixture was then added to 200 µL of lysis buffer (MALDI Sepsityper KIT, Bruker, Bremen, Germany). The preparations from the anaerobic bottles were treated the same way, except that no saponin was added. The samples were subsequently centrifuged at 13,000× *g* for two minutes and the supernatant removed. The pellet was resuspended in 1 mL of sterile MQ water. After an additional centrifugation at 13,000× *g* for two minutes, the supernatant was removed and the pellet resuspended in pre-warmed (37 °C) Müller Hinton Cation Adjusted Broth (MHCA, Sigma-Aldrich, Saint Louis, MO, USA) to McFarland 1.0 before the analysis (i.e., at the late exponential growth phase). 

### 4.4. Incubation Time and Antibiotic Concentration

The incubation time for the bacterial strains in the assay, with and without antibiotics, was set to 90 min. This threshold is based on previous findings, which demonstrated that this duration is sufficient for the reliable detection of growth differences under these conditions [21,22,28,29,30]. The antibiotic concentrations in the wells, which had a total volume of 200 µL, were set to either 64 mg/L of cefotaxime, 32 mg/L of meropenem, or 16 mg/L of ciprofloxacin in order to exclude indeterminant resistance. 

### 4.5. Assay Performance

The microplates were prepared with ten isolates and one positive and one negative control strain, with and without three different antibiotics, and in duplicate. All isolates were re-run if the duplicate results were inconclusive (in terms of resistant or sensitive). The absorbance was measured at 600 nm at the beginning of the assay (time: 0 min) and every ten minutes until 90 min of incubation at 37 °C with agitation in an Infinite 200 Pro plate reader (Tecan, Männedorf, Switzerland). The data from the reader were transferred to Excel (Microsoft 365), where the data evaluation was performed.

In order to approve the test, bacterial cultures without antibiotics (growth controls) had to grow above a threshold after 90 min of incubation. This growth threshold was set to an increase in absorbance of ≥50% based on previous findings [21,22,28,29,30]. Second, the absorbance value of each suspension after 90 min of incubation (*X*_90_) with an added antibiotic was compared to the initial (*X*_0_) absorbance in the same well, and the susceptibility/resistance was calculated according to the following formulae:

$$•\;\;\;\;\;\mathrm{Susceptible}:\;\frac{X_{90}}{1.5}\leq X_{0}$$

$$•\;\;\;\;\;\mathrm{Resistant}:\;\frac{X_{90}}{1.5}>X_{0}$$

The obtained results (susceptible/resistant) were compared to the previously achieved results from the E-test or disc diffusion tests described above. Differences between the assay and the routine test were expressed as a Major error (false resistance) or Very major error (false susceptibility). Sensitivity was defined as the number of true resistant isolates over the total number of resistant isolates. Specificity was defined as the number of true susceptible isolates over the total number of susceptible isolates. Accuracy was defined as the number of true resistant and true susceptible isolates over the total number of replicates.

## 5. Conclusions

We have, in this study, demonstrated a simple, quick, and highly accurate assay that can be used for the analysis of both isolated pure bacterial colonies and positive blood cultures. The method is based on the use of a common microplate reader, which is part of the basic equipment of nearly all microbiology laboratories globally. The assay can easily be performed, using three antibiotics, on 100 pure isolates in a day. In addition, it can be used to test a variety of other antibiotics and/or antibiotic concentrations, and the incubation time can easily be adjusted. 

## Figures and Tables

**Table 1:** Complete susceptibility analysis of all used strains and species, performed with the absorbance method, compared with the results from routine diagnostic tests. ABS = absorbance at 600 nm.

Antibiotic	Isolate Resistant by Routine Test		Isolates Susceptible by Routine Test		Total Analysis	Very Major Error Rate (%)	Major Error Rate (%)	Sensitivity (%)	Specificity (%)	Overall Accuracy (%)
	Susceptible by ABS	Resistant by ABS	Susceptible by ABS	Resistant by ABS						
Cefotaxim	4	638	353	9	1004	0.4	0.9	98.6	98.9	98.7
Meropenem	0	206	783	4	993	0.0	0.4	98.1	100.0	99.6
Ciprofloxacin	3	349	631	16	999	0.3	1.6	95.6	99.5	98.1
Overall	7	1193	1767	29	2996	0.2	1.0	97.6	99.6	98.8

**Table 2:** Susceptibility analysis of the control strains and the clinical and environmental strains, performed using the absorbance method, compared with the results from routine diagnostic tests. ABS = absorbance at 600 nm.

**Environmental and Clinical Isolates**										
**Antibiotic**	**Isolate Resistant by Routine Test**		**Isolates Susceptible by Routine Test**		**Total Analysis**	**Very Major Error Rate (%)**	**Major Error Rate (%)**	**Sensitivity (%)**	**Specificity (%)**	**Overall Accuracy (%)**
	**Susceptible by ABS**	**Resistant by ABS**	**Susceptible by ABS**	**Resistant by ABS**						
Cefotaxime	4	514	261	7	786	0.5	0.9	98.7	98.5	98.6
Meropenem	0	95	678	3	776	0.0	0.4	96.9	100.0	99.6
Ciprofloxacin	3	236	529	14	782	0.4	1.8	94.4	99.4	97.8
Overall	7	845	1468	24	2344	0.3	1.0	97.2	99.5	98.7
**Control Strains**										
**Antibiotic**	**Isolate Resistant by Routine Test**		**Isolates Susceptible by Routine Test**		**Total Analysis**	**Very Major Error rate (%)**	**Major Error Rate (%)**	**Sensitivity (%)**	**Specificity (%)**	**Overall Accuracy (%)**
	**Susceptible by ABS**	**Resistant by ABS**	**Susceptible by ABS**	**Resistant by ABS**						
Cefotaxime	0	124	92	2	218	0.0	0.9	98.4	100.0	99.1
Meropenem	0	111	105	1	217	0.0	0.5	99.1	100.0	99.5
Ciprofloxacin	0	113	102	2	217	0.0	0.9	98.3	100.0	99.1
Overall	0	348	299	5	652	0.0	0.8	98.6	100.0	99.2

**Table 3:** Resistance pattern of the different control strains used in this study.

Source	Number	Species	Resistance Genes	MIC by Etest		
				CTX (mg/L)	MER (mg/L)	CIP (mg/L)
CCUG	10785	*K. pneumoniae*	---	0.015	0.015	0.002
CCUG	8619400	*E. coli*	---	0.06	0.03	0.015
CCUG	17620	*E. coli*	---	0.094	0.012	0.015
CCUG	58538	*E. coli*	MOX	128	0.06	0.03
CCUG	58543	*E. coli*	CMY-2	64	0.03	0.06
CCUG	58547	*K. pneumoniae*	VIM	>256	>32	>32
CCUG	59351	*E. coli*	CTX-M 15	>256	0.015	>32
CCUG	59357	*E. coli*	SHV12/5A	3	0.008	0.03
CCUG	59360	*K. pneumoniae*	SHV12/5A	2	0.015	0.03
CMRS	100978	*E. coli*	SHV	2	0.008	0.015
CMRS	100979	*K. pneumoniae*	NDM, CTX-M1(15)	32	4	>32
CMRS	100980	*E. coli*	DHA	2	0.015	0.12
CMRS	100981	*K. pneumoniae*	DHA, CTX-M1(15)	>256	0.015	0.5
CMRS	100982	*K. pneumoniae*	KPC	>256	>32	>32
CMRS	100983	*K. pneumoniae*	KPC, CTX-M1(15), CMY-2	>256	>32	>32

## Data Availability

The data can be shared upon request. These data will become part of an upcoming thesis.
